# *catena*-Poly[[diaqua­manganese(II)]-μ-7-oxabicyclo­[2.2.1]heptane-2,3-dicarboxyl­ato]

**DOI:** 10.1107/S1600536810019021

**Published:** 2010-05-29

**Authors:** Na Wang, Jie Feng, Dong-Hang Wang, Qiu-Yue Lin

**Affiliations:** a Zhejiang Key Laboratory for Reactive Chemistry on Solid Surfaces, Institute of Physical Chemistry, Zhejiang Normal University, Jinhua, Zhejiang 321004, People’s Republic of China; b College of Chemistry and Life Science, Zhejiang Normal University, Jinhua 321004, Zhejiang, People’s Republic of China

## Abstract

In the title polymer, [Mn(C_8_H_8_O_5_)(H_2_O)_2_]_*n*_, the Mn^II^ atom is in a distorted octa­hedral coordination mode, binding to the bridging O atom of the bicyclo­heptane unit, two O atoms from corresponding carboxyl­ate groups, one carboxyl­ate O atom from a symmetry-related bridging ligand and two water O atoms. This arrangement leads to the formation of polymeric chains propagating parallel to [001]. The crystal structure is stabilized by several O—H⋯O hydrogen-bonding inter­actions involving the coordinated water mol­ecules as donors and the carboxyl­ate O atoms as acceptors.

## Related literature

7-Oxabicyclo­[2.2.1]heptane-2,3-dicarb­oxy­lic anhydride (nor­can­tharidin) is a lower toxicity anti­cancer drug, see: Shimi *et al.* (1982 ▶). For the preparation of disodium demethyl­cantharate, see: Yin *et al.* (2003 ▶).
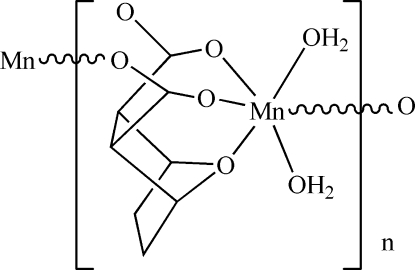


## Experimental

### 

#### Crystal data

[Mn(C_8_H_8_O_5_)(H_2_O)_2_]
*M* *_r_* = 275.12
Orthorhombic, 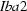

*a* = 10.3513 (2) Å
*b* = 18.9903 (4) Å
*c* = 10.4899 (5) Å
*V* = 2062.04 (11) Å^3^
*Z* = 8
Mo *K*α radiation
μ = 1.30 mm^−1^
*T* = 296 K
0.37 × 0.22 × 0.14 mm

#### Data collection

Bruker APEXII area-detector diffractometer
Absorption correction: multi-scan (*SADABS*; Sheldrick, 1996 ▶) *T*_min_ = 0.722, *T*_max_ = 0.838
14198 measured reflections
2401 independent reflections
2196 reflections with *I* > 2σ(*I*)
*R*_int_ = 0.030

#### Refinement

*R*[*F* ^2^ > 2σ(*F* ^2^)] = 0.024
*wR*(*F* ^2^) = 0.057
*S* = 1.02
2401 reflections
157 parameters
7 restraints
H atoms treated by a mixture of independent and constrained refinement
Δρ_max_ = 0.21 e Å^−3^
Δρ_min_ = −0.28 e Å^−3^
Absolute structure: Flack (1983 ▶), 1171 Friedel pairs
Flack parameter: 0.019 (15)

### 

Data collection: *APEX2* (Bruker, 2006 ▶); cell refinement: *SAINT* (Bruker, 2006 ▶); data reduction: *SAINT*; program(s) used to solve structure: *SHELXS97* (Sheldrick, 2008 ▶); program(s) used to refine structure: *SHELXL97* (Sheldrick, 2008 ▶); molecular graphics: *SHELXTL* (Sheldrick, 2008 ▶); software used to prepare material for publication: *SHELXL97*.

## Supplementary Material

Crystal structure: contains datablocks I, global. DOI: 10.1107/S1600536810019021/wm2346sup1.cif

Structure factors: contains datablocks I. DOI: 10.1107/S1600536810019021/wm2346Isup2.hkl

Additional supplementary materials:  crystallographic information; 3D view; checkCIF report

## Figures and Tables

**Table 1:** Selected bond lengths (Å)

Mn1—O3^i^	2.0910 (13)
Mn1—O4	2.1527 (13)
Mn1—O2*W*	2.1722 (16)
Mn1—O1*W*	2.1892 (13)
Mn1—O2	2.1929 (15)
Mn1—O1	2.2660 (11)

Symmetry code: (i) 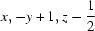.

**Table 2:** Hydrogen-bond geometry (Å, °)

*D*—H⋯*A*	*D*—H	H⋯*A*	*D*⋯*A*	*D*—H⋯*A*
O1*W*—H1*WA*⋯O4^ii^	0.87 (2)	1.83 (2)	2.6965 (17)	175 (3)
O2*W*—H2*WA*⋯O5^iii^	0.88 (2)	1.95 (2)	2.796 (2)	161 (3)
O1*W*—H1*WB*⋯O5^i^	0.82 (2)	2.01 (2)	2.809 (2)	166 (3)
O2*W*—H2*WB*⋯O2^i^	0.80 (2)	2.13 (2)	2.822 (2)	145 (3)

Symmetry codes: (i) 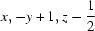; (ii) 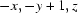; (iii) 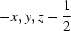.

**Fig. 1.**
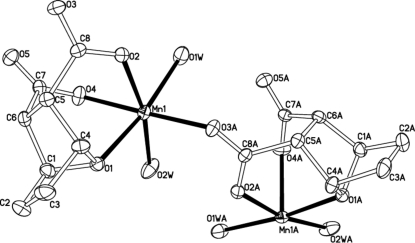
A view of the molecule of (I) showing the atom-labelling scheme with displacement ellipsoids drawn at the 30% probability.

## supplementary crystallographic information

### Comment

7-oxabicyclo[2,2,1]heptane-2,3-dicarboxylic anhydride (norcantharidin) derived from cantharidin is a lower toxicity anticancer drug (Shimi *et al.*, 1982) which makes this compound and its derived metal complexes interesting for structural research.

In the title polymer, each Mn^II^ atom is six-coordinated in a distorted octahedral coordination, defined by the bridging oxygen atom (O1) of the bicycloheptane unit, two oxygen atoms (O2 and O4) from carboxylate groups, one carboxylate oxygen atom (O3A) from a symmetry-related bridging ligand, and two oxygen atoms (O1W and O2W) from two water molecules. Each demethylcantharate anion adopts simultaneously a bridging coordination mode (O2 and O3 towards neighbouring Mn^II^ atoms) and a monodentate coordination mode (through O4 towards Mn1). Owing to the binding of the bridging oxygen atom (O1) with Mn^II^, two six-membered rings(Mn1/O2/C8/C5/C4/O1 and Mn1/O4/C7/C6/C1/O1) are created. In addition, a seven-membered ring (Mn1/O2/C8/C5/C6/C7/O4) is formed because of the coordination of the carboxylate oxygen atoms (O2 and O4).

The crystal structure is stabilized by several hydrogen bonding interactions of the type O—H···O, involving the coordinated water molecules as donors and carboxylate O atoms as acceptors (Table 2).

### Experimental

Disodium demethylcantharate was prepared in accordance with the literature technique (Yin *et al.*, 2003).

A solution of manganese(II) acetate (1 mmol) and disodium demethylcantharate (1 mmol) was stirred at the room temperature. After 1 h, a solution of 2-amino-1,3,4-thiadiazole (2 mmol) was added dropwise to the mixed solution. Crystals suitable for single-crystal X-ray diffraction were obtained as colourless blocks over a period of several weeks.

### Refinement

H atoms bonded to C atoms were positioned geometrically and were refined using a riding model [d(C—H) = 0.97-0.98 Å, U_iso_(H) = 1.2U_eq_(C)]. H atoms bonded to water O atoms were located in a difference Fourier maps and were refined with an O—H distance restraint of 0.85 (2) Å and Uiso(H) = 1.5Ueq(O).

### Crystal data

**Table:**

[Mn(C_8_H_8_O_5_)(H_2_O)_2_]	*F*(000) = 1128
*M**_r_* = 275.12	*D*_x_ = 1.772 Mg m^−^^3^
Orthorhombic, *I**b**a*2	Mo *K*α radiation, λ = 0.71073 Å
Hall symbol: I 2 -2c	Cell parameters from 6907 reflections
*a* = 10.3513 (2) Å	θ = 2.1–27.8°
*b* = 18.9903 (4) Å	µ = 1.30 mm^−^^1^
*c* = 10.4899 (5) Å	*T* = 296 K
*V* = 2062.04 (11) Å^3^	Block, colourless
*Z* = 8	0.37 × 0.22 × 0.14 mm

### Data collection

**Table:**

Bruker APEXII area-detector diffractometer	2401 independent reflections
Radiation source: fine-focus sealed tube	2196 reflections with *I* > 2σ(*I*)
graphite	*R*_int_ = 0.030
ω scans	θ_max_ = 27.8°, θ_min_ = 2.1°
Absorption correction: multi-scan (*SADABS*; Sheldrick, 1996)	*h* = −13→12
*T*_min_ = 0.722, *T*_max_ = 0.838	*k* = −23→24
14198 measured reflections	*l* = −13→13

### Refinement

**Table:**

Refinement on *F*^2^	Secondary atom site location: difference Fourier map
Least-squares matrix: full	Hydrogen site location: inferred from neighbouring sites
*R*[*F*^2^ > 2σ(*F*^2^)] = 0.024	H atoms treated by a mixture of independent and constrained refinement
*wR*(*F*^2^) = 0.057	*w* = 1/[σ^2^(*F*_o_^2^) + (0.0349*P*)^2^] where *P* = (*F*_o_^2^ + 2*F*_c_^2^)/3
*S* = 1.02	(Δ/σ)_max_ = 0.001
2401 reflections	Δρ_max_ = 0.21 e Å^−^^3^
157 parameters	Δρ_min_ = −0.28 e Å^−^^3^
7 restraints	Absolute structure: Flack (1983), 1171 Friedel pairs
Primary atom site location: structure-invariant direct methods	Flack parameter: 0.019 (15)

### Special details

**Table:**

Geometry. All esds (except the esd in the dihedral angle between two l.s. planes) are estimated using the full covariance matrix. The cell esds are taken into account individually in the estimation of esds in distances, angles and torsion angles; correlations between esds in cell parameters are only used when they are defined by crystal symmetry. An approximate (isotropic) treatment of cell esds is used for estimating esds involving l.s. planes.
Refinement. Refinement of *F*^2^ against ALL reflections. The weighted *R*-factor wR and goodness of fit *S* are based on *F*^2^, conventional *R*-factors *R* are based on *F*, with *F* set to zero for negative *F*^2^. The threshold expression of *F*^2^ > σ(*F*^2^) is used only for calculating *R*-factors(gt) etc. and is not relevant to the choice of reflections for refinement. *R*-factors based on *F*^2^ are statistically about twice as large as those based on *F*, and *R*- factors based on ALL data will be even larger.

### Fractional atomic coordinates and isotropic or equivalent isotropic displacement parameters (Å^2^)

**Table:**

	*x*	*y*	*z*	*U*_iso_*/*U*_eq_	
Mn1	0.23964 (2)	0.466637 (12)	0.21088 (5)	0.02642 (8)	
O1	0.37728 (10)	0.37412 (6)	0.22354 (12)	0.0264 (3)	
O3	0.35327 (13)	0.46639 (7)	0.59926 (14)	0.0309 (3)	
C8	0.35590 (15)	0.45297 (10)	0.48293 (17)	0.0235 (4)	
O1W	0.12217 (13)	0.56135 (8)	0.23773 (15)	0.0428 (4)	
C6	0.28402 (16)	0.32972 (9)	0.4091 (2)	0.0266 (4)	
H6A	0.2856	0.2879	0.4636	0.032*	
O5	0.08680 (12)	0.35748 (7)	0.51658 (13)	0.0365 (3)	
O2	0.32170 (11)	0.49510 (7)	0.39659 (14)	0.0300 (3)	
O4	0.11371 (12)	0.40095 (7)	0.32282 (15)	0.0359 (3)	
C5	0.40058 (15)	0.37989 (9)	0.44426 (17)	0.0250 (4)	
H5A	0.4519	0.3589	0.5130	0.030*	
C7	0.15121 (15)	0.36557 (9)	0.41784 (18)	0.0255 (4)	
O2W	0.15899 (16)	0.41688 (10)	0.04225 (15)	0.0490 (4)	
C4	0.47817 (16)	0.37949 (10)	0.31993 (18)	0.0289 (4)	
H4A	0.5344	0.4206	0.3094	0.035*	
C1	0.32042 (18)	0.30945 (10)	0.27210 (19)	0.0309 (4)	
H1A	0.2471	0.2926	0.2215	0.037*	
C3	0.54794 (18)	0.30928 (12)	0.3051 (2)	0.0394 (5)	
H3A	0.5904	0.2955	0.3837	0.047*	
H3B	0.6112	0.3111	0.2370	0.047*	
C2	0.4359 (2)	0.25889 (11)	0.2721 (2)	0.0439 (5)	
H2A	0.4481	0.2373	0.1892	0.053*	
H2B	0.4262	0.2224	0.3361	0.053*	
H2WA	0.0832 (18)	0.4022 (14)	0.017 (3)	0.066*	
H1WA	0.0462 (18)	0.5712 (14)	0.268 (2)	0.066*	
H2WB	0.193 (2)	0.4298 (16)	−0.022 (2)	0.066*	
H1WB	0.125 (2)	0.5871 (13)	0.176 (2)	0.066*	

### Atomic displacement parameters (Å^2^)

**Table:**

	*U*^11^	*U*^22^	*U*^33^	*U*^12^	*U*^13^	*U*^23^
Mn1	0.02597 (12)	0.02875 (14)	0.02454 (14)	−0.00026 (9)	0.00046 (14)	0.00452 (15)
O1	0.0268 (5)	0.0279 (6)	0.0246 (7)	0.0008 (4)	0.0014 (5)	−0.0003 (6)
O3	0.0305 (6)	0.0387 (7)	0.0235 (7)	0.0061 (5)	−0.0003 (5)	−0.0052 (5)
C8	0.0159 (7)	0.0306 (9)	0.0240 (10)	−0.0027 (6)	0.0019 (7)	−0.0023 (8)
O1W	0.0354 (7)	0.0479 (9)	0.0452 (10)	0.0157 (6)	0.0108 (6)	0.0147 (7)
C6	0.0284 (8)	0.0222 (8)	0.0292 (10)	−0.0010 (7)	0.0010 (7)	0.0041 (8)
O5	0.0320 (7)	0.0453 (8)	0.0321 (8)	0.0008 (6)	0.0049 (6)	0.0033 (6)
O2	0.0379 (7)	0.0265 (6)	0.0257 (7)	0.0034 (6)	−0.0002 (6)	0.0008 (6)
O4	0.0233 (6)	0.0449 (8)	0.0395 (9)	0.0013 (6)	0.0017 (6)	0.0161 (7)
C5	0.0218 (8)	0.0282 (9)	0.0250 (10)	0.0026 (7)	−0.0014 (7)	−0.0011 (8)
C7	0.0228 (8)	0.0243 (9)	0.0294 (10)	−0.0053 (7)	−0.0008 (7)	0.0013 (8)
O2W	0.0458 (9)	0.0724 (11)	0.0289 (8)	−0.0249 (8)	−0.0036 (7)	0.0029 (8)
C4	0.0210 (8)	0.0357 (10)	0.0301 (11)	0.0036 (7)	0.0013 (7)	−0.0052 (8)
C1	0.0345 (9)	0.0256 (9)	0.0325 (10)	−0.0025 (7)	−0.0015 (8)	−0.0038 (8)
C3	0.0342 (10)	0.0475 (12)	0.0365 (12)	0.0185 (9)	0.0023 (9)	−0.0050 (10)
C2	0.0553 (13)	0.0305 (11)	0.0461 (13)	0.0136 (9)	0.0063 (11)	−0.0045 (10)

### Geometric parameters (Å, °)

**Table:**

Mn1—O3^i^	2.0910 (13)	C6—H6A	0.9800
Mn1—O4	2.1527 (13)	O5—C7	1.241 (2)
Mn1—O2W	2.1722 (16)	O4—C7	1.263 (2)
Mn1—O1W	2.1892 (13)	C5—C4	1.532 (2)
Mn1—O2	2.1929 (15)	C5—H5A	0.9800
Mn1—O1	2.2660 (11)	O2W—H2WA	0.875 (16)
O1—C1	1.454 (2)	O2W—H2WB	0.800 (17)
O1—C4	1.457 (2)	C4—C3	1.524 (3)
O3—C8	1.247 (2)	C4—H4A	0.9800
O3—Mn1^ii^	2.0910 (13)	C1—C2	1.534 (3)
C8—O2	1.259 (2)	C1—H1A	0.9800
C8—C5	1.518 (2)	C3—C2	1.543 (3)
O1W—H1WA	0.867 (15)	C3—H3A	0.9700
O1W—H1WB	0.815 (16)	C3—H3B	0.9700
C6—C1	1.534 (3)	C2—H2A	0.9700
C6—C7	1.537 (2)	C2—H2B	0.9700
C6—C5	1.581 (2)		
			
O3^i^—Mn1—O4	176.92 (5)	C4—C5—C6	101.44 (14)
O3^i^—Mn1—O2W	91.42 (6)	C8—C5—H5A	109.9
O4—Mn1—O2W	87.68 (6)	C4—C5—H5A	109.9
O3^i^—Mn1—O1W	83.39 (5)	C6—C5—H5A	109.9
O4—Mn1—O1W	93.99 (5)	O5—C7—O4	123.99 (16)
O2W—Mn1—O1W	104.40 (7)	O5—C7—C6	118.40 (16)
O3^i^—Mn1—O2	97.45 (5)	O4—C7—C6	117.60 (16)
O4—Mn1—O2	83.84 (6)	Mn1—O2W—H2WA	137.1 (18)
O2W—Mn1—O2	168.38 (6)	Mn1—O2W—H2WB	113 (2)
O1W—Mn1—O2	84.17 (6)	H2WA—O2W—H2WB	103 (2)
O3^i^—Mn1—O1	98.67 (5)	O1—C4—C3	101.98 (14)
O4—Mn1—O1	84.23 (4)	O1—C4—C5	102.44 (13)
O2W—Mn1—O1	87.25 (6)	C3—C4—C5	109.84 (16)
O1W—Mn1—O1	168.16 (6)	O1—C4—H4A	113.8
O2—Mn1—O1	84.00 (5)	C3—C4—H4A	113.8
C1—O1—C4	96.10 (13)	C5—C4—H4A	113.8
C1—O1—Mn1	114.89 (9)	O1—C1—C2	102.31 (15)
C4—O1—Mn1	115.91 (9)	O1—C1—C6	102.45 (14)
C8—O3—Mn1^ii^	133.21 (12)	C2—C1—C6	110.39 (16)
O3—C8—O2	124.60 (17)	O1—C1—H1A	113.5
O3—C8—C5	117.07 (16)	C2—C1—H1A	113.5
O2—C8—C5	118.31 (16)	C6—C1—H1A	113.5
Mn1—O1W—H1WA	136.7 (17)	C4—C3—C2	102.08 (15)
Mn1—O1W—H1WB	112.0 (19)	C4—C3—H3A	111.4
H1WA—O1W—H1WB	100.8 (19)	C2—C3—H3A	111.4
C1—C6—C7	112.76 (16)	C4—C3—H3B	111.4
C1—C6—C5	100.51 (14)	C2—C3—H3B	111.4
C7—C6—C5	113.69 (14)	H3A—C3—H3B	109.2
C1—C6—H6A	109.8	C1—C2—C3	101.40 (15)
C7—C6—H6A	109.8	C1—C2—H2A	111.5
C5—C6—H6A	109.8	C3—C2—H2A	111.5
C8—O2—Mn1	126.25 (12)	C1—C2—H2B	111.5
C7—O4—Mn1	123.55 (11)	C3—C2—H2B	111.5
C8—C5—C4	113.07 (15)	H2A—C2—H2B	109.3
C8—C5—C6	112.38 (13)		
			
O3^i^—Mn1—O1—C1	−167.13 (12)	C1—C6—C5—C4	1.19 (16)
O4—Mn1—O1—C1	11.83 (12)	C7—C6—C5—C4	−119.54 (17)
O2W—Mn1—O1—C1	−76.11 (12)	Mn1—O4—C7—O5	141.81 (15)
O1W—Mn1—O1—C1	93.7 (3)	Mn1—O4—C7—C6	−39.0 (2)
O2—Mn1—O1—C1	96.22 (12)	C1—C6—C7—O5	149.40 (16)
O3^i^—Mn1—O1—C4	82.04 (11)	C5—C6—C7—O5	−97.02 (19)
O4—Mn1—O1—C4	−99.00 (11)	C1—C6—C7—O4	−29.8 (2)
O2W—Mn1—O1—C4	173.06 (12)	C5—C6—C7—O4	83.8 (2)
O1W—Mn1—O1—C4	−17.1 (3)	C1—O1—C4—C3	56.75 (16)
O2—Mn1—O1—C4	−14.61 (11)	Mn1—O1—C4—C3	178.24 (11)
Mn1^ii^—O3—C8—O2	27.8 (3)	C1—O1—C4—C5	−56.96 (15)
Mn1^ii^—O3—C8—C5	−150.61 (12)	Mn1—O1—C4—C5	64.53 (14)
O3—C8—O2—Mn1	−150.12 (13)	C8—C5—C4—O1	−86.60 (16)
C5—C8—O2—Mn1	28.30 (19)	C6—C5—C4—O1	33.96 (16)
O3^i^—Mn1—O2—C8	−133.89 (13)	C8—C5—C4—C3	165.60 (15)
O4—Mn1—O2—C8	48.92 (13)	C6—C5—C4—C3	−73.84 (17)
O2W—Mn1—O2—C8	5.5 (4)	C4—O1—C1—C2	−56.40 (16)
O1W—Mn1—O2—C8	143.59 (13)	Mn1—O1—C1—C2	−178.67 (11)
O1—Mn1—O2—C8	−35.89 (13)	C4—O1—C1—C6	58.02 (14)
O2W—Mn1—O4—C7	131.49 (16)	Mn1—O1—C1—C6	−64.25 (15)
O1W—Mn1—O4—C7	−124.24 (15)	C7—C6—C1—O1	85.26 (17)
O2—Mn1—O4—C7	−40.55 (15)	C5—C6—C1—O1	−36.13 (16)
O1—Mn1—O4—C7	44.01 (15)	C7—C6—C1—C2	−166.38 (15)
O3—C8—C5—C4	−144.55 (16)	C5—C6—C1—C2	72.23 (17)
O2—C8—C5—C4	36.9 (2)	O1—C4—C3—C2	−35.23 (18)
O3—C8—C5—C6	101.34 (18)	C5—C4—C3—C2	72.87 (18)
O2—C8—C5—C6	−77.21 (19)	O1—C1—C2—C3	34.25 (19)
C1—C6—C5—C8	122.23 (15)	C6—C1—C2—C3	−74.20 (19)
C7—C6—C5—C8	1.5 (2)	C4—C3—C2—C1	0.64 (19)

Symmetry codes: (i) *x*, −*y*+1, *z*−1/2; (ii) *x*, −*y*+1, *z*+1/2.

### Hydrogen-bond geometry (Å, °)

**Table:**

*D*—H···*A*	*D*—H	H···*A*	*D*···*A*	*D*—H···*A*
O1W—H1WA···O4^iii^	0.87 (2)	1.83 (2)	2.6965 (17)	175 (3)
O2W—H2WA···O5^iv^	0.88 (2)	1.95 (2)	2.796 (2)	161 (3)
O1W—H1WB···O5^i^	0.82 (2)	2.01 (2)	2.809 (2)	166 (3)
O2W—H2WB···O2^i^	0.80 (2)	2.13 (2)	2.822 (2)	145 (3)

Symmetry codes: (iii) −*x*, −*y*+1, *z*; (iv) −*x*, *y*, *z*−1/2; (i) *x*, −*y*+1, *z*−1/2.
